# Molecular Identification and Genotyping of *Enterocytozoon bieneusi* in Sheep in Shanxi Province, North China

**DOI:** 10.3390/ani12080993

**Published:** 2022-04-12

**Authors:** Rui-Lin Qin, Ya-Ya Liu, Jin-Jin Mei, Yang Zou, Zhen-Huan Zhang, Wen-Bin Zheng, Qing Liu, Wen-Wei Gao, Shi-Chen Xie, Xing-Quan Zhu

**Affiliations:** 1 College of Veterinary Medicine, Shanxi Agricultural University, Jinzhong 030801, China; q540604070@163.com (R.-L.Q.); ly1397772866@163.com (Y.-Y.L.); mjj3051@163.com (J.-J.M.); zzh13834778507@163.com (Z.-H.Z.); wenbinzheng1@126.com (W.-B.Z.); lqsxau@163.com (Q.L.); sxndgaowenwei@163.com (W.-W.G.); 2 Heilongjiang Key Laboratory for Zoonosis, College of Veterinary Medicine, Northeast Agricultural University, Harbin 150030, China; zouyangdr@163.com; 3 Research Center for Parasites & Vectors, College of Veterinary Medicine, Hunan Agricultural University, Changsha 410128, China; 4 Key Laboratory of Veterinary Public Health of Higher Education of Yunnan Province, College of Veterinary Medicine, Yunnan Agricultural University, Kunming 650201, China

**Keywords:** *Enterocytozoon bieneusi*, prevalence, genotypes, sheep, Shanxi Province

## Abstract

**Simple Summary:**

*Enterocytozoon bieneusi* is a fungus-like protist that is distributed worldwide and can cause malabsorption and diarrhea in sheep, other animals, and humans, threatening the development of animal husbandry and public health. However, little is known of the prevalence and genotypes of *E. bieneusi* in sheep in Shanxi Province, North China. Thus, 492 fecal samples were collected from sheep in three representative counties in northern, central, and southern Shanxi Province, and nested PCR amplification targeting the internal transcribed spacer (ITS) region of the rRNA gene was carried out to detect the prevalence and identify the genotypes of *E. bieneusi.* The overall *E. bieneusi* prevalence in the examined sheep in Shanxi Province was 34.2% (168/492). Four known genotypes (BEB6, COS-I, CHS7, and CHC8) and one novel genotype (SY-1) were detected, and the prevalent genotype was BEB6. The five genotypes observed in this study belong to the host-adapted Group 2. This is the first report on the occurrence and genotypes of *E. bieneusi* in sheep in Shanxi Province, which enhances an understanding of the global distribution and genetic diversity of *E. bieneusi* and has implications for the better control of *E. bieneusi* in animals and humans.

**Abstract:**

*Enterocytozoon bieneusi* is a fungus-like protist that can cause malabsorption and diarrhea in sheep, other animals, and humans, threatening the development of animal husbandry and public health. To date, there are no data about the prevalence and genotypes of *E. bieneusi* in sheep in Shanxi Province, North China. In this study, 492 fecal samples were collected from sheep in three representative counties in northern, central, and southern Shanxi Province. Nested PCR amplification was performed to detect the prevalence and identify the genotypes of *E. bieneusi* based on the internal transcribed spacer (ITS) region of the rRNA gene. Overall, 168 of 492 examined samples were *E. bieneusi*-positive, with a prevalence of 34.2% (168/492). Significant differences in the prevalence of *E. bieneusi* were observed among the three sampled regions (χ2 = 95.859, df = 2, *p* < 0.001), but the differences in *E. bieneusi* prevalence were not statistically significant between different genders and age groups (*p* > 0.05). Sequence analysis showed that four known genotypes (BEB6, COS-I, CHS7, and CHC8) and one novel genotype (named SY-1) were identified. BEB6 was the prevalent genotype found within the three counties. Phylogenetic analysis revealed that the five genotypes observed in this study belong to Group 2. The present study reported the presence and genotypes of *E. bieneusi* infection in sheep in Shanxi Province for the first time, which enriches the knowledge of the genetic diversity of *E. bieneusi* and provides baseline data for the prevention and control of *E. bieneusi* infection in animals and humans.

## 1. Introduction

Microsporidia are a class of obligate intracellular parasites that have been detected in most invertebrates and vertebrate hosts. Over 220 genera and approximately 1700 species have now been described [1]. Among them, 17 species have been frequently reported in humans [2], of which the most common and important species is *E. bieneusi* [3]. *E. bieneusi* was first isolated and identified in the intestinal epithelial cells of AIDS patients in 1985 [4]. *E. bieneusi* can cause symptomatic and asymptomatic intestinal infections. It can also cause acute diarrhea, poor absorption, respiratory inflammation, non-calculus cholecystitis, and even life-threatening diarrhea in individuals with immunodeficiency [5]. Moreover, *E. bieneusi* could lead to asymptomatic infections in people with normal immune status and transmit from infected individuals to susceptible humans or animals via a fecal-oral route [1]. Although animals and water are the potential reservoirs of *E. bieneusi* transmission, its public health risks have always been neglected due to its scarce incidence rate in humans.

Up to now, over 500 genotypes of *E. bieneusi* have been identified based on sequences of the internal transcribed spacer (ITS) region of the ribosomal DNA [6], and all genotypes can be allocated into 11 distinct phylogenetic groups (Groups 1 to 11). Group 1 is the largest group that comprises the majority of genotypes isolated from various hosts, including humans, and Group 2 was previously considered to consist of genotypes found in ruminants, but some of these genotypes are also found in humans [7], such as genotype I, J, BEB4, and BEB6 [8]. Groups 3–11 are host adaptation groups and might be present in specific hosts and wastewater [9]. At present, all the genotypes of *E. bieneusi* detected in sheep belonged to Groups 1, 2, and 7 [7].

Sheep are among the important food-producing animals, and over 8 million sheep are being raised in Shanxi Province, North China, annually. However, little is known of the prevalence, genetic diversity, and population genetic structure of *E. bieneusi* in sheep in Shanxi Province. Thus, the objectives of the present study were to investigate the prevalence and identify the genotypes of *E. bieneusi* in sheep in Shanxi Province, North China, which will enhance an understanding of the global genetic diversity of *E. bieneusi* and provide baseline data for executing measures for the prevention and control of *E. bieneusi* infection.

## 2. Materials and Methods

### 2.1. Specimen Collection

In November 2020, a total of 492 fecal samples were randomly collected from sheep in Qi County (*n* = 97), Shanyin County (*n* = 135), and Jishan County (*n* = 260) in central, northern, and southern Shanxi Province, respectively. All the sheep were intensively raised on farms and fed with grass. All fecal samples were separately packed in sterile tubes and loaded into foam boxes with ice packs. Then, these samples were transferred to the laboratory and stored in freezer at −20 °C until extraction of genomic DNA. Information of all samples was recorded, such as number, region, and age.

### 2.2. DNA Extraction and PCR Amplification

The genomic DNA of each fecal sample was extracted using the E.Z.N.A.^®^ Stool DNA Kit (Omega Bio-Tek Inc., Norcross, GA, USA) according to the manufacturer’s protocols. Then, the genomic DNA was stored at −20 °C prior to PCR amplification. The *E. bieneusi* positivity in sheep was determined by nested PCR amplification of ~389 bp fragment of the ITS rRNA gene. The outer primers for the primary amplification were F1 (5′-GGTTAGTAGTAGAGAGAGAG-3′) and R1 (5′-TTCGAGTTCTCGCGCTC-3′), and the inner primers for the second PCR amplification were F2 (5′-GCTCTATATCTATGGCT-3′) and R2 (5′-ATCGCGGAGAGACAAGTG-3′), as reported in a previous study [10]. The PCR reaction mixture (25 μL) consisted of 14.875 μL of ddH_2_O, 2 μL of dNTP Mix (2.5 mM each), 2.5 μL of 10× PCR Buffer, 2 mM of MgCl_2_ (25 mM), 0.625 U of r-*Taq* (TaKaRa Bio Inc., Tokyo, Japan), 0.25 μM of each primer, and approximately 150 ng of the genomic DNA. The first PCR product was used as the template for secondary PCR amplification. The PCR conditions were as follows: initial denaturation at 94 °C for 5 min, 35 cycles of denaturation at 94 °C for 45 s, annealing at 55 °C (primary PCR and secondary PCR) for 45 s, and extension at 72 °C for 1 min; this was followed by a final extension at 72 °C for 10 min. The secondary PCR products were analyzed by electrophoresis in 1.5% agarose gels containing ethidium bromide, and the secondary positive products were sent to Sangon Biotech Co. Ltd. (Shanghai, China) for bidirectional sequencing.

### 2.3. Sequencing and Phylogenetic Analysis

The obtained sequences were revised and aligned by Chromas V2.6 and Basic Local Alignment Search Tool (BLAST), respectively, to identify the genotypes of *E. bieneusi*. The phylogenetic tree was constructed using neighbor-joining (NJ) method with 1000 bootstraps in MEGA7 software. The novel genotypes were identified according to the established nomenclature system [11]. All obtained representative sequences in this study were deposited in GenBank with the accession numbers OL604153 to OL604157.

### 2.4. Statistical Analysis

All statistical analyses in this study were performed using the software SPSS 26.0 (IBM, Chicago, IL, USA) for data processing. Chi-square (χ2) test was used to calculate the significant differences in *E. bieneusi* among different regions, ages, and genders. Odds ratios (ORs) and 95% confidence intervals (95% CIs) were used to assess the reliability of results among prevalence and test conditions. Results were considered statistically significant when *p* value < 0.05.

## 3. Results

### 3.1. Prevalence of E. bieneusi in Sheep

In this study, 168 samples tested *E. bieneusi*-positive, and the overall prevalence of *E. bieneusi* in sheep in Shanxi Province was 34.15% (168/492). The *E. bieneusi* infection existed in each examined county and all age groups (Table 1), and there were significant differences in *E. bieneusi* prevalence among the different counties (*p* < 0.001). Moreover, the highest prevalence of *E. bieneusi* was detected in Qi County (74.2%), followed by Jishan County (29.6%) and Shanyin County (14.1%). Between the two age groups, the prevalence of *E. bieneusi* in lamb (<6 month) was 36.0%, which was higher than that in older sheep (>6 months) (32.7%), but the difference was not statistically significant (*p* = 0.448).

### 3.2. Genotype Distribution of E. bieneusi in Sheep

**Figure 1:**
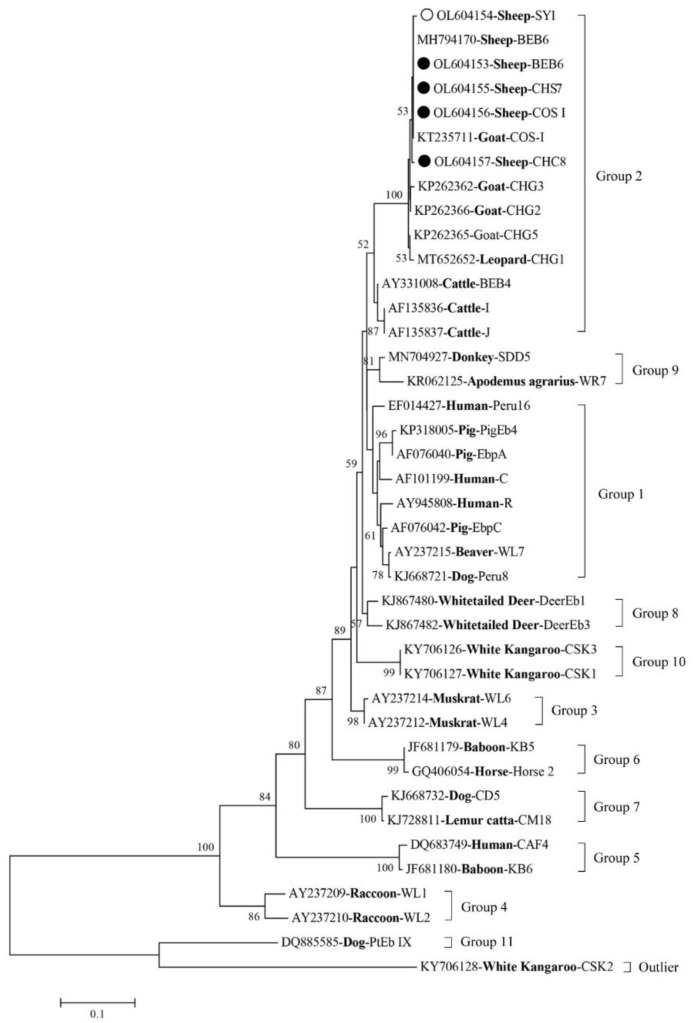
Phylogenetic relationships of four known genotypes (marked with black circle) and one novel genotype (marked with unfilled circle) of *Enterocytozoon bieneusi*, along with other genotypes identified in this study and previous reports. Bootstrap value higher than 50% is shown.

Among the 168 *E. bieneusi*-positive samples, 4 known genotypes (BEB6, COS-I, CHS7, and CHC8) and 1 novel genotype (designated SY-1) were identified (Table 2). Of these genotypes, BEB6 sequences (*n* = 135), COS-I sequences (*n* = 23), CHS7 sequences (*n* = 2), and a CHC8 sequence (*n* = 1) showed a 100% similarity to MN728943 (BEB6), KX383622 (COS-I), KP262392 (CHS7), and MK139946 (CHC8), respectively. Genotype BEB6 was the dominant genotype distributed in Shanyin County (52.63%, 10/19), Jishan County (68.83%, 53/77), and Qi County (100%, 72/72). The phylogenetic tree revealed that all five genotypes were clustered into the host-adapted Group 2 (Figure 1).

## 4. Discussion

Prior to the present study, little was known regarding the prevalence and genotypes of *E. bieneusi* in sheep in Shanxi Province, North China. The present study revealed that the overall prevalence of *E. bieneusi* in sheep in Shanxi Province was 34.15% (168/492), which was higher than that reported in sheep in many other Chinese counties and provinces but lower than that compared with some other counties and provinces in China (Table 3). There are many factors influencing the prevalence reported in these studies, such as herd size, sheep breed, sample collection area, age, feeding habits, and hygiene conditions. Measures need to be taken to prevent and control the infection of *E. bieneusi* in sheep. For example, animals infected with parasites should be kept separately from healthy animals and treated with albendazole or fumagillin [12], disinfected regularly, and ventilated properly [13].

Shanxi Province has a temperate continental monsoon climate with a mountain area of 80%, and basins and valleys are the principal locations for residents [33]. Among the three investigation regions considered in the present study, the difference in the prevalence of *E. bieneusi* was statistically significant (*p* < 0.001). As shown in Table 2, the highest *E. bieneusi* prevalence was observed in Farm 1 to Farm 4 in Qi County. Qi County is close to the city of Taiyuan, which is the trade center and transportation hub in Shanxi Province. Thus, we reasoned that the specific geographical location and the high mobilization of persons might be directly or indirectly responsible for the higher *E. bieneusi* prevalence in Qi County; thus, more samples should be examined in the future to further clarify the possible reasons explaining *E. bieneusi* prevalence in these regions.

The incomplete immunity of young sheep and living in a susceptible environment may be the causes of the high risk of *E. bieneusi* infection [13], which may also be the reasons why the higher prevalence was observed in young sheep in this study.

In this study, four known *E. bieneusi* genotypes (BEB6, COS-I, CHS7, and CHC8) and one novel genotype (SY-1) were identified. Among these genotypes, BEB6, COS-I, CHS7, and CHC8 have commonly been identified in ruminants worldwide [14,25,27,28]. The BEB6 (135/170, 79.4%) genotype was the predominant genotype found within the three study regions, which is consistent with what was found in sheep in the Inner Mongolia Autonomous Region [24]. BEB6 (*n* = 72) was also the only genotype found in sheep in Qi County, which suggests its importance and zoonotic potential in this local area. With its host range expansion, BEB6 is currently considered to be a human genotype with extensive geographical distribution [22]. In addition, the BEB6 genotype has been identified in deer [34], goats [13], wild and domestic animals, and humans [7]. Moreover, the COS-I (synonym: CM7) genotype has been identified in humans, NHPs, and domestic and wild animals [5,7]; and the genotypes CHS7 and CHC8 have been identified in domestic animals, such as cattle, sheep, and goats in China [5]. Phylogenetic analysis showed that all five genotypes identified in the present study were clustered into the host-adapted Group 2, indicating that sheep may be a source of *E. bieneusi* infection for humans and other animals.

## 5. Conclusions

The present study revealed an *E. bieneusi* prevalence of 34.15% in sheep in Shanxi Province, North China. Significant differences in the prevalence of *E. bieneusi* were found among the different study regions. ITS sequencing identified four known genotypes (BEB6, COS-I, CHS7, and CHC8) and one novel genotype (SY-1) in the sheep, with BEB6 being the zoonotic genotype distributed in all three examined counties. This is the first documentation of *E. bieneusi* prevalence and genotypes in sheep in Shanxi Province, which enriched the knowledge of the geographical distribution and genetic diversity of *E. bieneusi* and provided baseline data for the better control of *E. bieneusi* in animals and humans.

## Figures and Tables

**Table 1:** Factors associated with prevalence of *Enterocytozoon bieneusi* in sheep in Shanxi Province.

Factor	Categories	No. Examined	No. Positive	Prevalence % (95%CI)	OR (95%CI)	*p* Value
Region	Qi	97	72	74.2 (65.5–82.9)	17.6 (9.0–34.2)	*p* < 0.001
	Shanyin	135	19	14.1 (8.2–19.9)	1	
	Jishan	260	77	29.6 (24.1–35.2)	2.6 (1.5–4.5)	
Age	≤6	211	76	36.0 (29.5–42.5)	1.2 (0.8–1.7)	0.448
	>6	281	92	32.7 (27.3–38.2)	1	

**Table 2:** Prevalence and genotypes of *Enterocytozoon bieneusi* in sheep in Shanxi Province.

Region	Farm ID	No. Positive/Examined	Prevalence %	Genotype (*n*)
Qi	Farm 1	15/21	71.4	BEB6 (15)
	Farm 2	33/38	86.8	BEB6 (33)
	Farm 3	18/29	62.1	BEB6 (18)
	Farm 4	6/9	66.7	BEB6 (6)
Shanyin	Farm 5	14/100	14.0	BEB6 (7); SY-1 (6); CHS7 (1)
	Farm 6	3/21	14.3	BEB6 (1); CHS7 (1); COS-I (1)
	Farm 7	0/8	0	-
	Farm 8	2/6	33.3	BEB6 (2)
Jishan	Farm 9	29/92	31.5	BEB6 (6); COS-I (22); SY-1 (1)
	Farm 10	38/130	29.2	BEB6 (37); CHC8 (1)
	Farm 11	4/8	50.0	BEB6 (4)
	Farm 12	0/7	0	-
	Farm 13	6/23	26.1	BEB6 (6)
Total		168/492		BEB6 (135), COS-I (23), SY-1 (7), CHS7 (2), CHC8 (1)

**Table 3:** Prevalence of *Enterocytozoon bieneusi* in sheep worldwide.

Country	District	No. Positive/Total	Prevalence (%)	Gene Locus	Years	Reference
Sweden		49/109	45.0	ITS	2014	[14]
Iran	Tehran	3/30	10.0	SSU rRNA	2012–2013	[15]
Brazil	Rio de Janeiro	24/125	19.2	ITS	2016	[16]
Egypt	Giza	6/89	6.7	SSU rRNA	2012–2013	[17]
Slovakia	Eastern	2/45	4.4	ITSSSU rRNA	2012	[18]
China	Qinghai	16/38	42.1	ITS	2018	[19]
		73/312	23.4	ITS	2013–2015	[20]
		7/76	9.2	ITS	2012–2015	[21]
	Anhui	11/697	1.6	ITS	2015	[22]
		6/52	11.5	ITS	2012–2015	[21]
	Jiangsu	16/75	21.3	ITS	2015	[22]
	Shandong	1/60	1.6	ITS	2015	[22]
		16/122	13.1	ITS	2012–2015	[21]
	Xinjiang	20/318	6.3	ITS	2015–2017	[23]
		19/99	19.2	ITS	2012–2015	[21]
	Inner Mongolia	260/375	69.3	ITS	2015	[24]
		3/102	2.9	ITS	2012–2015	[21]
	Ningxia	148/360	41.1	ITS	2016–2017	[25]
		124/1014	12.2	ITS	2019	[26]
		57/121	47.1	ITS	2012–2015	[21]
	Tibet	93/620	15.0	ITS	2016	[27]
	Henan	161/310	51.9	ITS	2011–2013	[28]
		0/35	0	ITS	2012–2015	[21]
	Liaoning	6/64	9.4	ITS	2011–2013	[28]
	Heilongjiang	10/40	25.0	ITS	2011–2013	[28]
		31/60	51.7	ITS	2012–2015	[21]
		31/138	22.5	ITS	2013–2014	[29]
		68/489	13.9	ITS	2013–2014	[30]
		2/45	4.4	ITS	2012	[31]
	Gansu	61/177	34.5	ITS	2015	[32]
	Shanghai	36/152	23.7	ITS	2012–2015	[21]
	Beijing	0/64	0	ITS	2012–2015	[21]
	Jilin	19/70	27.1	ITS	2012–2015	[21]
	Yunnan	40/325	12.3	ITS	2018	[13]

## Data Availability

The data sets supporting the results of this article have been submitted to the GenBank and the accession number is shown in the article.
